# Anti-apoptotic effects of Sonic hedgehog signalling through oxidative stress reduction in astrocytes co-cultured with excretory-secretory products of larval *Angiostrongylus cantonensis*

**DOI:** 10.1038/srep41574

**Published:** 2017-02-07

**Authors:** Kuang-Yao Chen, Cheng-Hsun Chiu, Lian-Chen Wang

**Affiliations:** 1 Department of Parasitology, College of Medicine, Chang Gung University, Taoyuan 333, Taiwan; 2 Molecular Infectious Disease Research Center, Chang Gung Memorial Hospital, Taoyuan, Taiwan; 3 Graduate Institute of Biomedical Sciences, College of Medicine, Chang Gung University, Taoyuan 333, Taiwan

## Abstract

*Angiostrongylus cantonensis*, the rat lungworm, is an important aetiologic agent of eosinophilic meningitis and meningoencephalitis in humans. Co-culturing astrocytes with soluble antigens of *A. cantonensis* activated the Sonic hedgehog (Shh) signalling pathway and inhibited the apoptosis of astrocytes via the activation of Bcl-2. This study was conducted to determine the roles of the Shh signalling pathway, apoptosis, and oxidative stress in astrocytes after treatment with excretory-secretory products (ESP) from *A. cantonensis* fifth-stage larvae. Although astrocyte viability was significantly decreased after ESP treatment, the expression of Shh signalling pathway related proteins (Shh, Ptch-1 and Gli-1) was significantly increased. However, apoptosis in astrocytes was significantly decreased after activation of the Shh signalling pathway. Moreover, superoxide and hydrogen superoxide levels in astrocytes were significantly reduced after the activation of Shh pathway signalling due to increasing levels of the antioxidants catalase and superoxide dismutase. These findings indicate that the anti-apoptotic effects of the Shh signalling pathway in the astrocytes of mice infected with *A. cantonensis* are due to reduced levels of oxidative stress caused by the activation of antioxidants.

The neurotropic characteristics of larval *Angiostrongylus cantonensis* can lead to eosinophilic meningitis or eosinophilic meningoencephalitis in humans. This parasite causes the infection of a human host upon ingesting snails, slugs, planarians or vegetables contaminated with the infective third-stage (L3) larvae. The larvae penetrate the intestinal wall and migrate to the central nervous system (CNS) via the circulation. They molt twice and develop into sexually immature fifth-stage larvae (L5)^1^,^2^,^3^,^4^. In the CNS, the larval stage parasites have been reported to induce Th2 immune responses^5^,^6^,^7^,^8^. Eosinophils infiltrate the subarachnoid space and cerebrospinal fluid (CSF), where they signal Th2 responses by producing Th2 cytokines, excreting chemoattractants, and presenting antigens to CD4^+^ T cells^9^. Moreover, blood-brain barrier (BBB) dysfunction is another feature of cerebral angiostrongyliasis and has been attributed to the activity of host matrix metalloproteinase-9^10^.

In a previous study, we demonstrated both BBB dysfunction and activation of astrocytes in mice infected with *A. cantonensis*. Moreover, the Sonic hedgehog (Shh) signalling pathway in astrocytes was found to be activated via the GRP78-dependent pathway after infection. Activation of this pathway significantly increased the survival of astrocytes^11^. It has been reported that Shh expressed in astrocytes is significantly higher than in neurons under oxidative stress^12^. The Shh pathway is also activated in reactive astrocytes and plays an important role in promoting cell proliferation during brain injury^13^,^14^. Under oxidative stress, secreted Shh may reduce apoptosis by activating Bcl-2 and inhibiting Bax through the PI3-K/AKT/Bcl-2 pathway^12^. These findings indicate an association between the Shh signalling pathway and oxidative stress.

Oxidative stress occurs when there is an imbalance between the production of reactive oxygen species (ROS) (H_2_O_2_, O2^−^ and OH^−^) and their subsequent neutralization^15^,^16^. It is an important pathological feature associated with the neurodegenerative diseases such as Alzheimer’s disease and Parkinson’s disease^17^,^18^,^19^. After infection with protozoan parasites such as *Plasmodium falciparum, Leishmania infantum, Toxoplasma gondii* and *Trypanosoma cruzi*, oxidative stress is induced in host cells, causing a wide range of effects, such as formation of DNA lesions and apoptosis^20^,^21^,^22^,^23^,^24^,^25^,^26^,^27^. However, oxidative stress and the expression of antioxidants such as glutathione reductase (GR), glutathione peroxidase (GPx) and glutathione S-transferase (GST) have both been found to be elevated in the CSF of mice infected with *A. cantonensis*^28^,^29^. Although oxidative stress may aggravate meningitis and brain injuries, antioxidants may reduce the levels of damage due to oxidative stress.

Excretory-secretory products (ESPs) are a valuable tool for the study of host-parasite relationships^30^. They contain a wide range of molecules that penetrate host defensive barriers and avoid the immune system of the host^31^. Recently, ESPs of adult *A. cantonensis* have been characterized and 17 proteins identified^32^. In the present study, we prepared and used ESP from L5 *A. cantonensis* to study the relationship between the Shh pathway, oxidative stress and apoptosis. The results indicate that oxidative stress and apoptosis in astrocytes are elevated after co-culture with ESP. However, the activation of Shh signalling pathway increased cell survival via inhibition of the generation of ROS and subsequent apoptosis. Moreover, the anti-apoptotic effects of Shh signalling are attributed to reduced oxidative stress levels.

## Results

### Identification of apoptotic effects of ESPs on astrocytes

**Figure 1:**
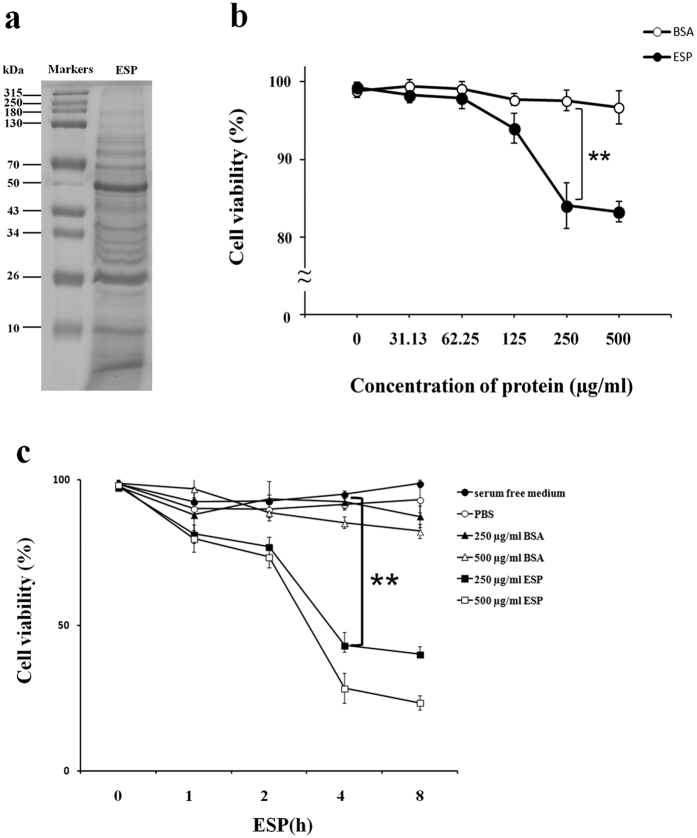
Collection of *Angiostrongylus cantonensis* excretory-secretory products (ESP) and induction of cell death in astrocytes. (**a**) ESP were obtained from the living fifth-stage larvae cultured in RPMI medium for 24, 48, 72 and 96 h. SDS-PAGE was used to determine the protein concentrations of ESP. (**b**) Viability of astrocytes in co-cultures with different concentrations of ESP (0 to 500 μg/ml) for 2 h and at (**c**) different time points (0 to 8 h). Bovine serum albumin (BSA) and PBS were used as controls (white circles). Data are expressed as the means ± SD based on three independent experiments (**P < 0.01).

ESPs were obtained from the culture medium of living L5 cultured in serum free RPMI media at 37 °C under 5% CO_2_ incubated for 96 h. Protein concentration was determined by SDS-PAGE with coomassie blue staining (Fig. 1a). The ESP preparations were found to induce dose- (Fig. 1b) and time-dependent (Fig. 1c) decreases in cell viability of astrocytes using a cell viability assay. The survival of astrocytes decreased to approximately 80% in the group treated with 500 μg/ml-ESP after 2 h (Fig. 1c).

**Figure 2:**
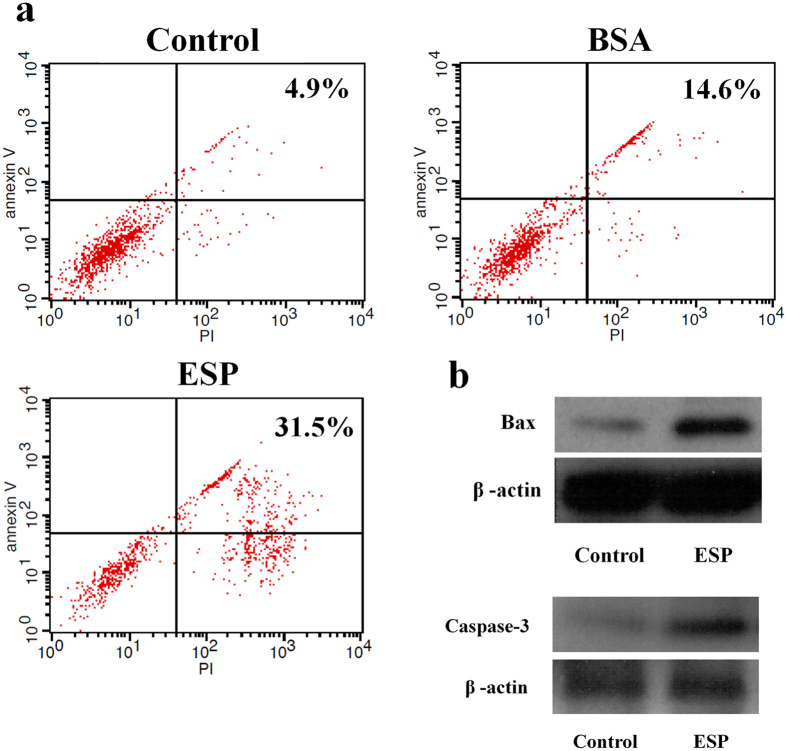
Apoptosis of astrocytes induced by *Angiostrongylus cantonensis* excretory-secretory products (ESP). (**a**) FASCan flow cytometric analysis of apoptosis of astrocytes co-cultured with or without 500 μg/ml ESP or BSA for 4 h (Annexin V−/PI−: normal cells, Annexin V+/PI−: early stage apoptotic cells, Annexin V+/PI+: late stage apoptotic cells, Annexin V−/PI+: necrosis cells). (**b**) Western blot analysis of Bax and Caspase-3 levels in astrocytes with and without treatment of 500 μg/ml ESP for 4 h. β-actin was used as a control. The full-length blots are presented in Supplementary Figures S1 and S2.

Apoptosis in astrocytes co-cultured with ESPs or bovine serum albumin (BSA) for 4 h was detected by an apoptosis assay. The percentage of late stage apoptotic cells (Annexin V+/PI+) in the ESP-treated group (31.5%) was higher than the BSA-treated group (14.6%) or the control group (4.9%) (Fig. 2a). Moreover, Western blot analysis also showed the elevation of apoptosis pathway upstream (Bax) and downstream (Caspase-3) proteins in the ESP-treated group (Fig. 2b). These findings suggest that ESPs induce the apoptosis of astrocytes.

### Activation of the Shh signalling pathway in ESP-treated astrocytes

**Figure 3:**
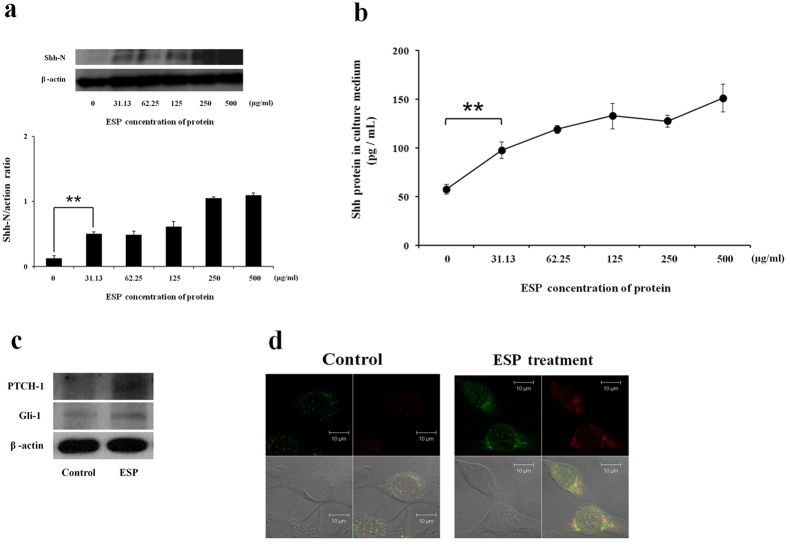
Activation of the Sonic hedgehog singlling (Shh) pathway in astrocytes co-cultured with *Angiostrongylus cantonensis* excretory-secretory products (ESP). (**a**) Western blot analysis of the expression level of Shh-N in astrocytes co-cultured with different concentrations of ESP for 4 h. β-actin is shown as a control. The full-length blots are presented in Supplementary Figure S3. Data are expressed as the means ± SD from three independent experiments (**P < 0.01). (**b**) Changes in the concentration of Shh protein in culture media of astrocytes co-cultured with ESP detected by a Shh ELISA kit after incubating for 4 h. Data are expressed as the means ± SD from three independent experiments (**P < 0.01). (**c**) Western blot analysis of Shh pathway downstream protein (PTCH-1 and Gli-1) levels in astrocytes incubated with ESP (500 μg/ml) for 4 h. β-actin is shown as a control. The full-length blots are presented in Supplementary Figure S4. (**d**) The expression levels of Shh and GFAP in astrocytes. The Shh and GFAP protein expression was determined by immunofluorescence staining of astrocytes with and without 500 μg/ml ESP treatment for 4 h (GFAP: green; Shh: red; co-localization of GFAP and Shh: yellow).

Western blot analysis revealed that the expression of Shh protein was significantly increased in astrocytes co-cultured with ESPs for 4 h (*P* < 0.01) (Fig. 3a). In addition, analysis of culture medium via ELISA demonstrated that secreted Shh was significantly elevated in the ESP-treated groups (*P* < 0.01) (Fig. 3b). Additional analysis of samples via Western blot demonstrated elevated expression levels of Ptch-1 and Gli-1, downstream Shh pathway proteins, after ESP treatment (Fig. 3c). Immunofluorescent staining for Shh and GFAP showed that Shh was mainly localized to the cytoplasm of activated astrocytes (Fig. 3d). The increased expression of Shh pathway related proteins in ESP-treated astrocytes indicates the activation of the Shh signalling pathway.

### Anti-apoptotic effects of the Shh signalling pathway in ESP-treated astrocytes

**Figure 4:**
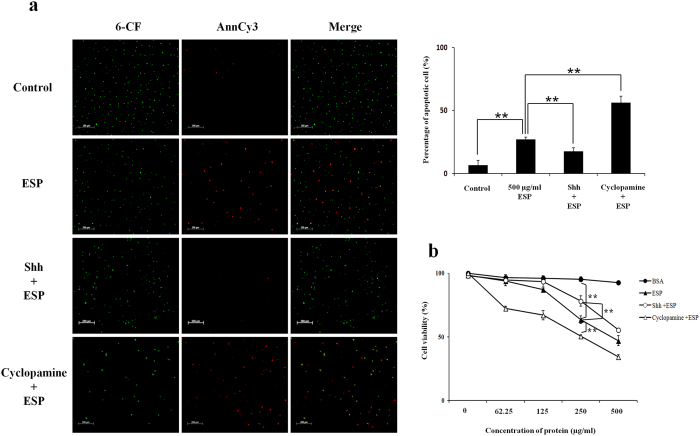
Anti-apoptotic effects of the Sonic hedgehog signalling pathway in astrocytes treated with *Angiostrongylus cantonensis* excretory-secretory products (ESP). Astrocytes were pre-treated with recombinant murine Sonic hedgehog peptide (Shh) (3 μg) or cyclopamine (20 μM) for 2 h. (**a**) Apoptosis was detected in pre-treated astrocytes co-cultured with 500 μg/ml ESP for 4 h using the Annexin V-Cy3 apoptosis assay. (**b**) Cell viability in co-cultures of the pre-treated astrocytes with different concentrations of ESP for 4 h determined using the CCK-8 assay. Data are expressed as the means ± SD from three independent experiments (**P < 0.01).

To determine the anti-apoptotic effects of the Shh signalling pathway, Shh was either downregulated using cyclopamine, a specific Shh inhibitor, or upregulated using a recombinant murine Shh peptide (rShh). Although apoptotic cells (6-CF + /Annexin V-Cy3 + cells) were detected in astrocytes treated with ESP alone (27.0%) and rShh with ESPs (17.3%), apoptosis in astrocytes treated with cyclopamine and ESPs was found to be significantly increased (56.0%) (*P* < 0.01) (Fig. 4a). In addition, the cell survival of the cyclopamine-treated group was significantly lower than other treatment groups (*P* < 0.01) (Fig. 4a).

### Inhibitory effects of the Shh signalling pathway on oxidative stress in ESP-treated astrocytes

After co-culturing astrocytes with ESPs alone, immunofluorescence staining demonstrated the expression of the oxidative stress marker superoxide (O_2_^−^) in the cells.

**Figure 5:**
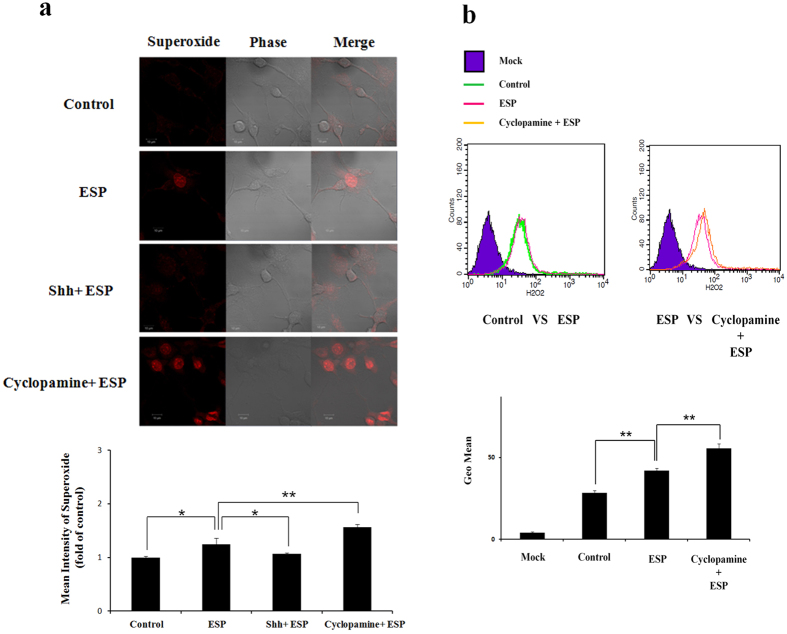
Reduction of superoxide (O_2_^−^) and H_2_O_2_ in astrocytes treated with *Angiostrongylus* cantonensis excretory-secretory products (ESP) via activation of the Sonic hedgehog singlling pathway. Astrocytes were pre-treated for 2 h with a recombinant murine Sonic hedgehog peptide (Shh) (3 μg) or cyclopamine (20 μM) and co-cultured with ESP (500 μg/ml) for 4 h. (**a**) The expression of superoxide was detected by dihydroethidium staining (excitation 535 nm, emission 610 nm). (**b**) The expression of H_2_O_2_ in astrocytes was detected by the H2DCFDA assay. Data are expressed as the means ± SD from three independent experiments (*P < 0.05, **P < 0.01).

**Figure 6:**
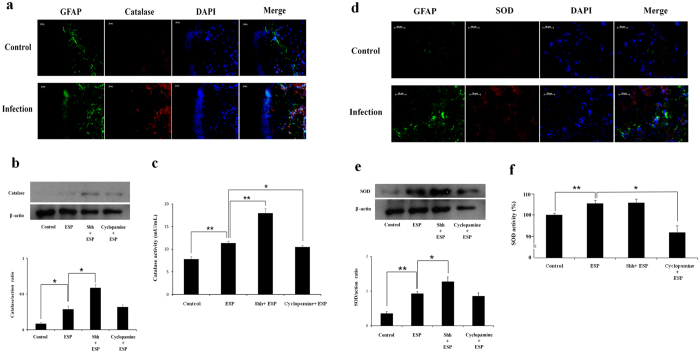
Activation of catalase and superoxide dismutase (SOD) in astrocytes treated with *Angiostrongylus cantonensis* excretory-secretory products (ESP) via activation of the Sonic hedgehog singlling pathway. (**a**) Fluorescent microscopic image demonstrated catalase in astrocytes of the hippocampus of BALB/c mice infected with 25 *A. cantonensis* third-stage larvae on day 28 post-infection (GFAP: green; Shh: red; co-localization of GFAP and Shh: yellow). (**b**) Western blot analysis on catalase in astrocytes treated with ESP alone, pre-treated with a recombinant Sonic hedgehog peptide from mouse (Shh) (3 μg) or cyclopamine (20 μM) for 2 h and then with 500 μg/ml ESP for 4 h. The full-length blots are presented in Supplementary Figure S5. (**c**) Activities of catalase determined by catalase activity assay. (**d**) Fluorescent photomicrographs showing SOD in the astrocytes from the hippocampus of infected BALB/c mice. (**e**) Western blot analysis on SOD in astrocytes. The full-length blots are presented in Supplementary Figure S6. (**f**) Activities of catalase determined by SOD activity assay. β-actin was used as a control. Data are expressed as means ± SD from three independent experiments (*P < 0.05, **P < 0.01).

**Figure 7:**
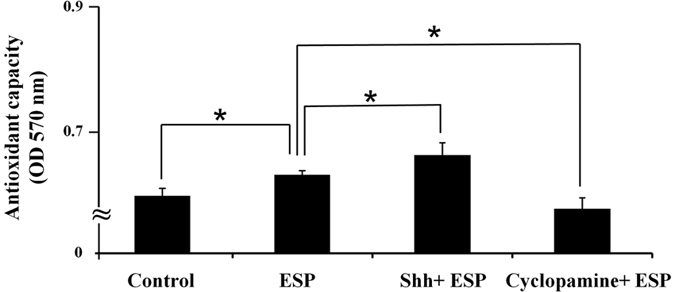
Elevation of antioxidant capacity in astrocytes treated with *Angiostrongylus cantonensis* excretory-secretory products (ESP) via activation of Sonic hedgehog singling pathway. Astrocytes were treated with ESP alone or pre-treated with a recombinant Sonic hedgehog peptide from mouse (Shh) (3 μg) or cyclopamine (20 μM) for 2 h and then treated with 500 μg/ml ESP for 4 h. Antioxidant capacity was then detected using the total antioxidant capacity (TAC) colorimetric assay kit. Data are expressed as the means ± SD from three independent experiments (*P < 0.05).

Pre-treatment with cyclopamine caused the increased expression of O_2_^−^ relative to ESP-only treated cells (*P* < 0.01) (Fig. 5a). Flow cytometric analysis revealed that ESP treatment increased the expression of H_2_O_2_ and that cyclopamine pre-treatment augmented this phenotype (*P* < 0.01) (Fig. 5b). The antioxidants catalase and superoxide dismutase (SOD) were found in astrocytes from the hippocampus of BALB/c mice infected with *A. cantonensis*, characterized using fluorescent microscopy (Fig. 6a and d). The levels (Fig. 6b and e) and activities (6c and 6 f) of these enzymes were significantly higher in cells treated with ESP alone than those pre-treated with cyclopamine (*P* < 0.05). Moreover, antioxidant capacity in astrocytes treated with ESP was also significantly elevated relative to those pre-treated with cyclopamine (*P* < 0.05) (Fig. 7). The presence of O_2_^−^ and H_2_O_2_ in the ESP-treated astrocytes suggests that they are under oxidative stress. However, activation of the Shh signalling pathway not only elevates the levels of the antioxidants but also increases their activities. These changes lead to increased antioxidant capacity.

## Discussion

It has been reported that ESPs from parasitic trematodes including *Fasciola hepatica*^33^,^34^,^35^, *Clonorchis sinensis*^36^ and *Paragonimus westermani*^37^,^38^ or cestodes like *Echinococcus multilocularis*^39^ may induce the apoptosis of host cells. In addition, blood-dwelling nematodes such as *Onchocerca volvulus*^40^ and *Brugia malayi*^41^ may interfere with the proliferation of host T cells and dendritic cells by the induction of apoptosis. In our previous study, apoptosis was detected in astrocytes treated with soluble antigen or living worms of *A. cantonensis,* and evaluated by detection of levels of apoptosis-related proteins Bax and Bcl-2^11^. The same phenomenon was observed in this study with cells co-cultured with ESP from L5 of *A. cantonensis*. However, activation of the Shh signalling pathway was found to reduce the level of apoptosis (Fig. 4a) and also elevate cellular survival rates (Fig. 4b).

Oxidative stress is caused by the generation of ROS in the living organisms and has been reported in cases of infection^42^,^43^,^44^ and cancer^45^,^46^,^47^. These highly reactive molecules, such as O_2_^−^ and H_2_O_2_, have been documented to cause apoptosis. Moreover, parasitic protozoa may also cause DNA lesions and apoptosis via oxidative stress^20^,^21^,^22^,^23^,^24^,^25^,^26^,^27^. Moreover, ROS have also been detected in CSF of mice infected with *A. cantonensis*^28^. After co-culturing with ESPs, the levels of superoxide and hydrogen peroxide in astrocytes significantly increased (Fig. 5). Moreover, these levels remained significantly high in astrocytes pre-treated with the specific Shh signal inhibitor cyclopamine but were significantly reduced in cells pre-treated with exogenous recombinant Shh protein. These findings indicate a role for the Shh signalling pathway in reducing oxidative stress.

**Figure 8:**
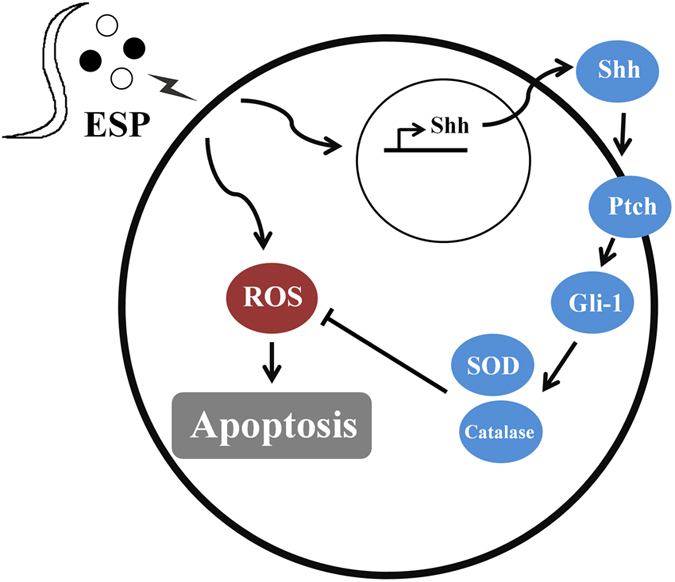
Schematic diagram of anti-apoptotic effects of Sonic hedgehog (Shh) signalling pathway in astrocytes co-cultured with excretory-secretory products (ESP) of *Angiostrongylus cantonensis* through oxidative stress reduction.

In conclusion, the present study demonstrates that the Shh signalling pathway has a significant protective effect against oxidative stress via increasing the expression and activity of antioxidants. Moreover, increased cell survival suggests an anti-apoptotic role of this pathway. Since Shh protects cortical neurons from apoptosis via a reduction of oxidative stress^48^, it is possible that this signalling pathway may also have apoptotic effects in the astrocytes of mice infected with *A. cantonensis* by reducing oxidative stress through the activation of antioxidants (Fig. 8).

## Material and Methods

### Animals and infection

The strain of *A. cantonensis* used has been maintained in our laboratory since 1980 by cycling growth in Sprague-Dawley (SD) rats and *Biomphalaria glabrata* snails^49^. SD rats and BALB/c mice were purchased from the National Laboratory Animal Center, Taipei. They were maintained in the Laboratory Animal Center according to guidelines approved by the Chang Gung University Institutional Animal Care and Use Committee (CGU12-157). Rats and mice were orally infected with 25 L3 isolated from infected snails.

### Preparation of ESP

After anaesthetizing with 30 μl of Zoletil 50 (Virbac, France), living L5 were isolated from the brain tissue of infected rats. L5 were washed with saline, phosphate buffered saline (PBS), distilled water, and RPMI containing high concentration of antimycotic solution (Sigma-Aldrich, USA) before incubating in RPMI without foetal bovine serum (FBS) for 96 h. The culture medium was concentrated using Amicon Ultra-15 10 K centrifugal filter devices (Merck Millipore, Germany). The collected material was used as EPS preparations, and protein concentrations were determined with the Bio-Rad Protein Assay Kit (Bio-Rad, Hercules, CA, USA).

### Cell culture

Mouse astrocytes (CRL-2535) were purchased from the American Type Culture Collection (ATCC). These cells were maintained in Dulbecco’s modified Eagle’s medium (Corning, USA) supplemented with 10% FBS and 100 U/ml penicillin/streptomycin. Aliquots (0.25 × 10^6^ cells/cm^2^) were then seeded on poly-L-lysine coated culture plates and cultured at 37 °C under 5% CO_2_ until they grew to a confluent layer of 1–2 × 10^4^ cells/cm^2^. By GFAP staining, over 95% of the cultured cells were identified to be astrocytes.

### Western blot analysis

Levels of the apoptosis-related proteins Bax, Bcl-2, GFAP, and Shh proteins were determined using a 12.5% SDS-PAGE. Proteins in the gels were transferred to nitrocellulose membrane using a semi-dry transfer unit at 0.04 mA for 50 min. The membrane was washed with TBS/T in triplicate and then with a blocking buffer. Primary antibodies employed include rabbit anti-Shh (1:100) (Santa Cruz Biotechnology Inc., USA), rabbit anti-Bax (1:500) (Santa Cruz Biotechnology Inc., USA), rabbit anti-Bcl-2 (1:500) (Santa Cruz Biotechnology Inc., USA), rabbit anti-GFAP (1:1,000) (Abcam, UK), rabbit anti-GRP78 (1:1,000) (Sigma-Aldrich, USA), and mouse anti-β-actin (1:5,000) (Sigma-Aldrich, USA). The membranes were incubated with these antibodies at 4 °C overnight. After washing three times TBS/T, the membranes were incubated with the corresponding secondary antibodies (goat anti-rabbit IgG 1:10,000 and rabbit anti-mouse IgG 1:10,000) (Sigma-Aldrich, USA) for 45 min and then washed with TBS/T in triplicate before incubating with a mixture of stable peroxide solution (500 μl) and enhanced solution (500 μl) in the dark for 5 min.

### ELISA

After incubating for 0–8 h, supernatants were collected from the co-cultures of ESP with astrocytes. Shh concentrations were detected using the mouse specific Shh-N ELISA kit (Sigma-Aldrich, USA).

### Immunohistochemistry and immunofluorescence staining

Brain tissues from BALB/c mice were snap-frozen by immersing in isopentane chilled to −70 °C and immediately mounted in OCT medium. The samples were then stored at −80 °C until sectioned by Microm OMV cryostat to 10–15 mm. The frozen tissue sections were fixed in 2% (w/v) paraformaldehyde (PFA) and permeabilized in 0.5% Triton X-100 in PBS before incubation with purified antibodies (Rabbit anti-Shh (1:50)) (Santa Cruz Biotechnology Inc., USA), rabbit anti-GFAP (1:500) (Abcam, UK), rabbit anti-SOD (1:1,000) (Sigma-Aldrich, USA) and rabbit anti-Catalase (1:1,000) (Sigma-Aldrich, USA) for 2 h at room temperature or overnight at 4 °C. After incubation, the sections were incubated with appropriate secondary antibodies (DyLightTM 488-594-conjugate anti-rabbit IgG) (1:1,000) (Jackson ImmunoResearch Inc, UK) for 1 h at room temperature. For nuclear counterstaining, the sections were stained with DAPI. The stained sections were then examined and photographed under a light microscope.

### Drug treatment

Prior to co-culturing experiments, cells were incubated in a serum-free DMEM for 12 h. Cells were treated with ESP (500 μg/ml) for 4 h. Groups pre-treated with cyclopamine (Sigma-Aldrich, USA) (20 μM) or rShh (Sigma-Aldrich, USA) (3 μg) for 2 h were also treated with 500 μg of ESPs for 4 h.

### Apoptosis assay

Cellular apoptosis was determined using the Annexin V- Cy3 apoptosis detection kit (Sigma-Aldrich, USA). Astrocytes from different drug treatment groups were washed twice with cold PBS and resuspended in 100 μl of binding buffer before incubating with 5 μl of Annexin V conjugated with FITC (Fluorescein isothiocyanate) and 10 μl of PI for 15 min at room temperature. Finally, each sample was washed five times with 50 μl of binding buffer. Samples resuspended in 35 μl of binding buffer were analysed by FASCan flow cytometer (BD Biosciences, USA).

### Cell viability assay

To determine cell survival, astrocytes (1 × 10^7^ cells/ml) were incubated with 50 ml of CCK-8 (Cell Counting Kit-8) (Sigma-Aldrich, USA) at 37 °C in the dark with mild shaking for 1 h. In the presence of cells, highly water-soluble tetrazolium salt WST-8 [2-(2-methoxy-4-nitrophenyl)-3-(4-nitrophenyl)-5-(2,4-disulfophenyl)-2H-tetrazolium, monosodium salt] produces a water-soluble formazan dye upon reduction. Cell survival is monitored by measuring formazan dye absorbance at 450 nm using a spectrophotometer (Molecular Devices, USA).

### Detection of ROS

Astrocytes removed from culture media by centrifugation were resuspended in a PBS buffer. To detect endogenous ROS production, control or ESP-treated astrocytes (1 × 10^7^ cells/ml) were incubated with 10 μM H2DCFDA (2′,7′-dichlorodihydrofluorescein diacetate) (Invitrogen., USA) at 26 °C in the dark with mild shaking for 30 min. ROS production was monitored using a spectrofluorometer (Molecular Devices, USA) by measuring DCF emission at 530 nm after excitation at 488 nm for 1 h.

### Catalase activity assay

Catalase activity in astrocytes was detected using the catalase activity colorimetric/fluorometric assay kit (BioVision, USA). The cells (10^6^) were incubated with 12 μl of fresh 1-mM H_2_O_2_ at 25 °C for 30 min, and 10 μl of stop solution was added. The samples were then incubated with 50 μl of Developer Mix (46 μl of assay buffer, 2 μl of OxiRed™ Probe and 2 μl HRP solution) at 25 °C for 10 min. Catalase activity was determined using a spectrofluorometer (Molecular Devices, USA) to measure the absorbance at 570 nm.

### Superoxide dismutase (SOD) activity assay

SOD activity in astrocytes was measured using the SOD determination kit (Sigma-Aldrich, USA) according to the manufacturer’s instructions. The activities were determined using a microplate reader to read the absorbance at 450 nm.

### Antioxidant capacity assay

The total antioxidant capacity (TAC) colorimetric assay kit (BioVision, USA) was used to detect antioxidant capacity in astrocytes. Control and ESP-treated astrocytes (1 × 10^7^ cells/ml) were incubated with 100 μl of Cu^2+^ for 1.5 h in the dark at room temperature with mild shaking. Antioxidant capacity was monitored using a spectrofluorometer (Molecular Devices, USA) to read the absorbance at 570 nm.

### Statistical analysis

The results are expressed as the means ± standard deviation (SD). Two-tailed Student’s *t*-test was performed for unpaired samples. *P* < 0.05 was considered statistically significant.

## Additional Information

**How to cite this article**: Chen, K.-Y. *et al*. Anti-apoptotic effects of Sonic hedgehog signalling through oxidative stress reduction in astrocytes co-cultured with excretory-secretory products of larval *Angiostrongylus cantonensis. Sci. Rep.* **7**, 41574; doi: 10.1038/srep41574 (2017).

**Publisher's note:** Springer Nature remains neutral with regard to jurisdictional claims in published maps and institutional affiliations.

## Supplementary Material

Supplementary Information
