# Supplementary material for: Anti-apoptotic effects of Sonic hedgehog signalling through oxidative stress reduction in astrocytes co-cultured with excretory-secretory products of larval Angiostrongylus cantonensis
Source: Sci Rep. 2017 Feb 7;7:41574. doi: 10.1038/srep41574 (PMC5294578; doi:10.1038/srep41574)
Supplement: Supplementary Information [file srep41574-s1.pdf]

Supplementary Information

Anti-apoptotic effects of Sonic hedgehog signalling through oxidative stress reduction in astrocytes co-cultured with excretory-secretory products of larval *Angiostrongylus cantonensis*

Kuang-Yao Chen[1], Cheng-Hsun Chiu[2] and Lian-Chen Wang[1, 2, 3, *]

[1] *Department of Parasitology, College of Medicine, Chang Gung University, Taoyuan 333, Taiwan*
[2]*Molecular Infectious Disease Research Center, Chang Gung Memorial Hospital, Taoyuan, Taiwan;*
[3]*Graduate Institute of Biomedical Sciences, College of Medicine, Chang Gung University, Taoyuan 333, Taiwan*

* Corresponding author. wanglc@mail.cgu.edu.tw

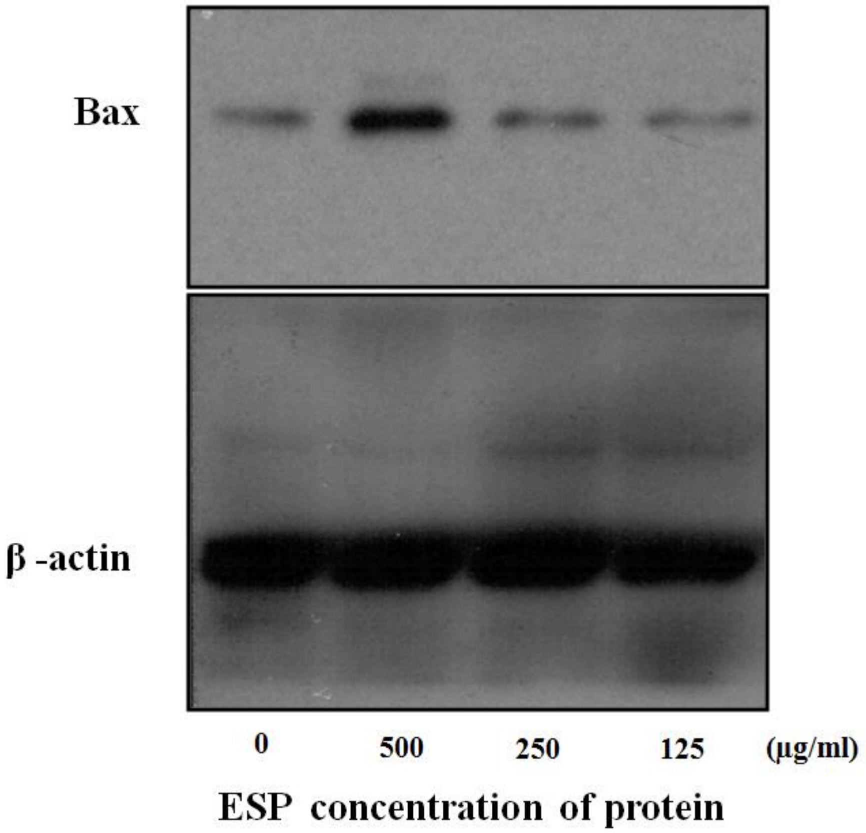

Figure S1. Western blot analysis of Bax level in astrocytes treated with different concentrations of ESP for 4 h. β-actin was used as a control.

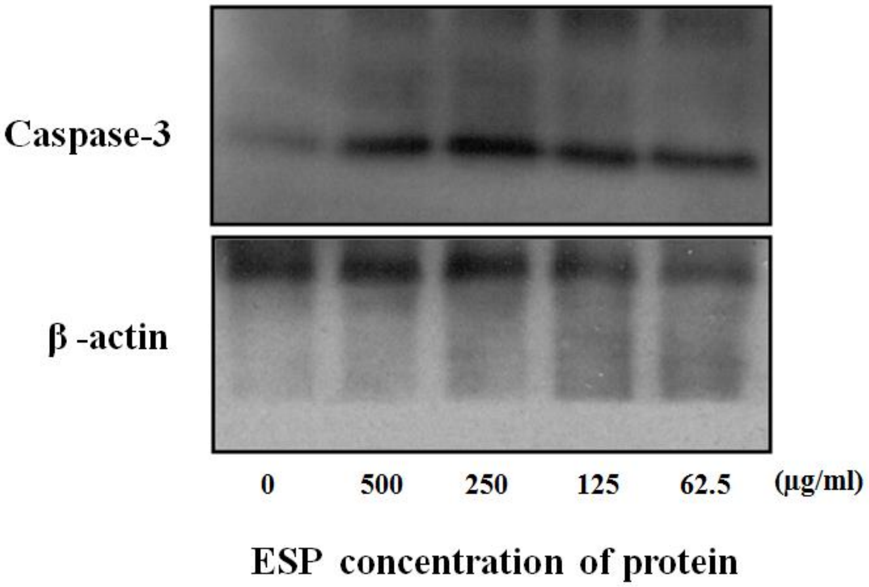

Figure S2. Western blot analysis of Caspase-3 level in astrocytes treated with different concentrations of ESP for 4 h. β-actin was used as a control.

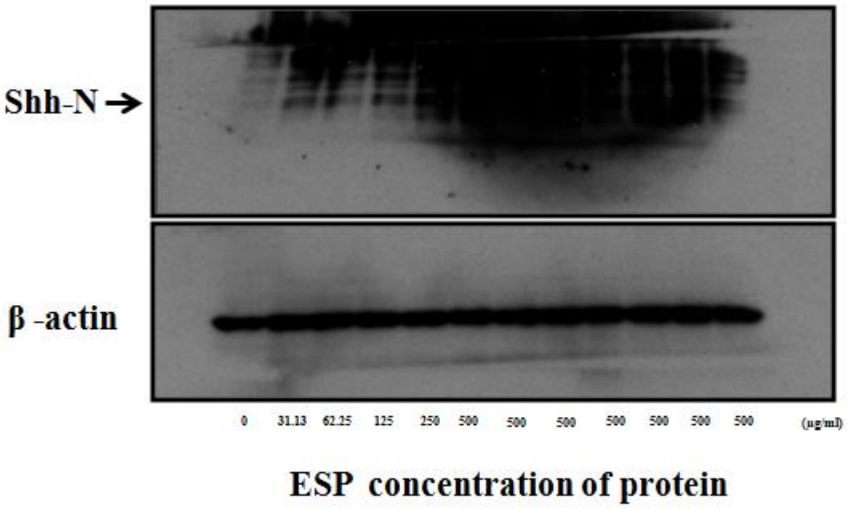

Figure S3. Western blot analysis of Shh level in astrocytes treated with different concentrations of ESP for 4 h. β-actin was used as a control.

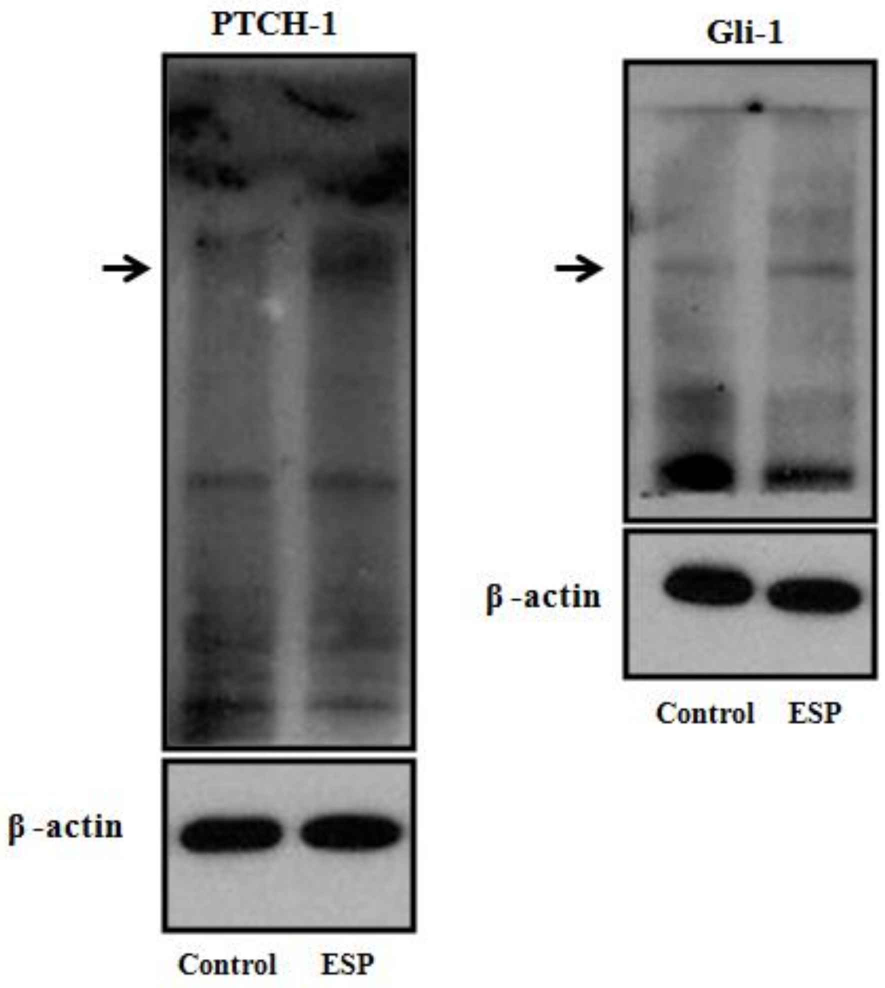

Figure S4. Western blot analysis of PTCH-1 and Gli-1 levels in astrocytes treated with or without 500 µg/ml ESP for 4 h. β-actin was used as a control.

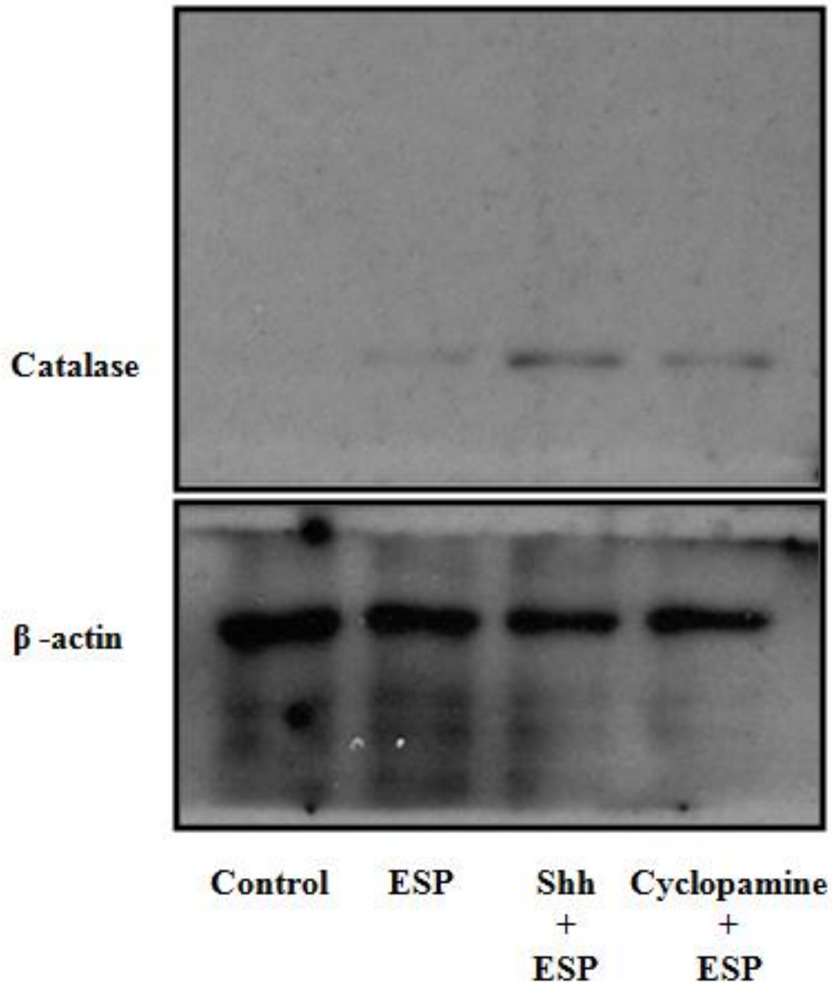

Figure S5. Western blot analysis on catalase in astrocytes treated with ESP alone, pre-treated with a recombinant Sonic hedgehog peptide from mouse (Shh) (3 µg) or cyclopamine (20 µM) for 2 h and then with 500 µg/ml ESP for 4 h. β-actin was used as a control.

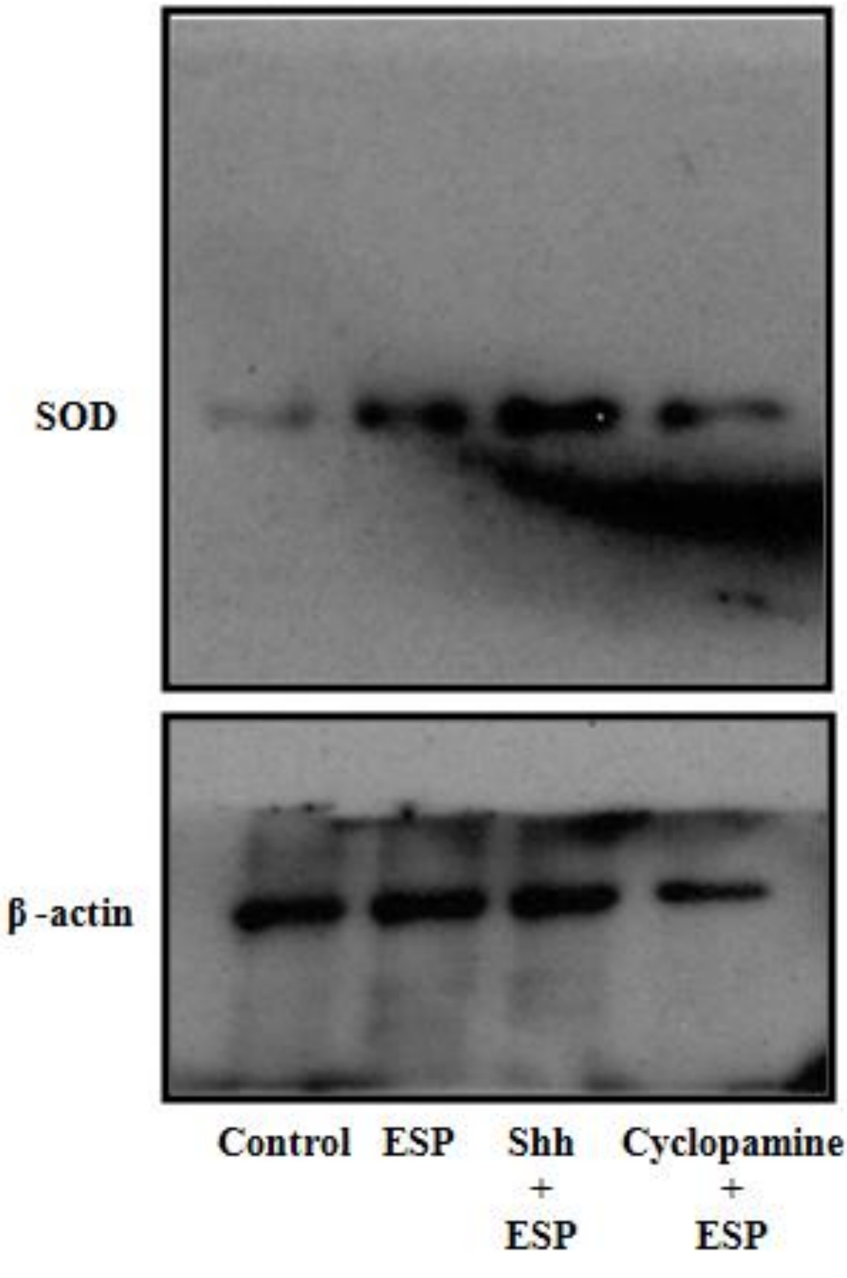

Figure S6. Western blot analysis on SOD in astrocytes treated with ESP alone, pre-treated with a recombinant Sonic hedgehog peptide from mouse (Shh) (3 µg) or cyclopamine (20 µM) for 2 h and then with 500 µg/ml ESP for 4 h. β-actin was used as a control.
